# Gonadotropin Stimulation Has Only a Limited Effect on the Concentration of Follicular Fluid Signalling Proteins: An Antibody Array Analysis

**DOI:** 10.1155/2021/2906164

**Published:** 2021-01-27

**Authors:** Nick A. Bersinger, Markus Eisenhut, Petra Stute, Michael von Wolff

**Affiliations:** University Women's Hospital, Division of Gynaecological Endocrinology and Reproductive Medicine, University of Berne, Switzerland

## Abstract

**Objective:**

The follicular fluid (FF) plays an essential role in the physiology of the follicle and the oocyte. Gonadotropin stimulation affects the FF steroid hormone and anti-Mullerian hormone (AMH) concentrations, which has been suggested to be the reason for lower oocyte competence in conventional gonadotropin stimulated *in vitro* fertilisation (cIVF) compared to natural cycle IVF (NC-IVF). To analyse the effect of gonadotropin stimulation on a broad spectrum of signalling proteins, we ran proteomic antibody arrays on FF of women undergoing both treatments NC-IVF and cIVF.

**Method:**

Twenty women underwent one NC-IVF and one cIVF treatment cycle. Follicular fluids of the first aspirated follicle were compared between the two groups using a protein microarray which included antibodies against 224 proteins related to cell signalling and reference proteins. Each of the 40 albumin-stripped, matched-pair samples was labelled in the reverse-dye (Cy3/Cy5) procedure before undergoing array hybridisation. Signal analysis was performed using normalisation algorithms in dedicated software. Five proteins yielding a value of *P* < 0.05 in the array experiment (Cystatin A, Caspase-3, GAD65/67, ERK-1, and ERK-2) were then submitted to quantitative determination by ELISA in the same follicular fluids.

**Results:**

Array analysis yielded only a small number of differentially expressed signalling markers by unadjusted *P* values. Adjustment as a consequence of multiple determinations resulted in the absence of any significant differential marker expression on the array. Five unadjusted differentially expressed proteins were quantified immunometrically with antibodies from different sources. Follicular fluid concentrations of Cystatin A and MAP kinase ERK-1 concentrations were significantly higher in the cIVF than in the NC-IVF follicles, while GAD-2 (GAD65/67) did not differ. The assays for Caspase-3 and MAP kinase ERK-2 did not have the required sensitivities.

**Conclusion:**

In contrast to FF steroid hormones and AMH, FF concentrations of signalling proteins are not or only marginally altered by gonadotropin stimulation.

## 1. Introduction

Follicular fluid (FF) is composed of different elements such as hormones, enzymes, anticoagulants, electrolytes, reactive oxygen species, and antioxidants [1]. It provides the oocyte with nutrients and mediates its communication with the follicle and thereby with the female endocrine system. In conventional in vitro fertilisation (cIVF) therapies, recruitment of the follicles is intensified by high dosages of exogenous gonadotropins, resulting in an unphysiological polyfollicular response. The gonadotropin stimulation has been suggested to be the reason for the lower oocyte competence in cIVF [2].

Indeed, several studies revealed that gonadotropin stimulation alters the concentration of several FF protein components such as cytokines including leukaemia inhibitory factor (LIF) [3–5], steroid hormones [3, 6], and anti-Mullerian hormone (AMH) [6, 7].

AMH is an FF marker for the implantation potential of the oocyte [8–10]. In cIVF follicles, AMH concentrations are reduced.

The cytokine LIF has been suggested to be a FF marker of the oocyte/embryo quality [11, 12]. In cIVF follicles, LIF concentrations are reduced [4].

These findings prompted us not to analyse regulatory proteins which are already well characterised in the context of follicular function but to widen the analysis and to screen the FF for a broad spectrum of signalling proteins using an antibody-based proteomic approach.

Such a protocol, using SDS-PAGE, OFFGEL, and SCX-based separation followed by LC-MS/MS analysis had already been performed by Ambekar et al. [13]. In six women, all undergoing cIVF, 480 proteins had been noted, of which 320 had not been described previously. The identified proteins belonged to diverse functional categories including growth factors and hormones, receptor signaling, enzyme catalysis, defense/immunity, and complement activity.

However, only a small number of women were analysed, and the study was not designed to evaluate the effect of gonadotropins.

To obtain the best possible match between the two treatment groups, we therefore selected a set of women who underwent both NC-IVF and cIVF with the intention to identify differences in a wide spectrum of signalling proteins.

## 2. Materials and Methods

### 2.1. Patients

Twenty women undergoing IVF treatment in the University Women's Hospital, Bern, Switzerland, between February 2012 and August 2015 for different infertility causes were enrolled in the study (Table 1). The principal inclusion condition was the availability of follicular fluid from a NC-IVF and from a cIVF treatment cycle for each of them. When several NC-IVF cycles with FF were available for the same patient, they were further matched by selecting the one with the closest patient age and sample storage time to the corresponding cIVF cycle.

### 2.2. IVF Procedure and Follicular Fluid Collection

In the NC-IVF protocol, cycles were monitored by serum luteinizing hormone (LH) and 17*β*-estradiol (E_2_) levels and by ultrasound. When the diameter of the follicle reached 18 mm or more, serum E_2_ concentration was above 800 pmol/L, 5,000 IU of hCG (Predalon®, MSD Merck Sharp & Dohme GmbH, Lucerne, Switzerland) was administered. The aspiration of the follicle and its contents was performed 36 hours later as described [14].

In the cIVF protocol, patients were given 150 to 300 IU of highly purified human menopausal gonadotrophin (Menopur HP®, Ferring AG, Baar, Switzerland) per day starting between days 3 and 5 of the menstrual cycle. The gonadotropin releasing hormone (GnRH) antagonist (Orgalutran®, Ganirelix 0.25 mg, MSD Merck Sharp & Dohme GmbH, Lucerne, Switzerland) was initiated between cycle days 6 and 7 and continued until the induction of ovulation. This was done after adequate follicular growth with 10,000 IU of urinary human chorionic gonadotrophin (hCG) (Pregnyl®, MSD Merck Sharp & Dohme GmbH, Lucerne, Switzerland). Transvaginal oocyte retrieval was performed 36 hours later under ultrasound guidance. The FF aspirated from the first follicle (≥18 mm) was collected separately.

The obtained single-follicle fluids were clarified after the removal of the oocyte and cumulus cells by centrifugation in two steps, first, at 600 x g for 10 min and then at 1300 x g for a further 10 min. The obtained supernatants were stored at -80°C until further processing for antibody arrays and hormone analyses.

Informed written consent was obtained prior to treatment, and the study was approved by the cantonal ethical committee Bern, Switzerland (KEK 2020-01682).

### 2.3. Antibody Arrays

The Panorama® Antibody Microarray–Cell Signalling Kit (Cat. No. CSAA1) from Sigma-Aldrich (St. Louis, USA) was used. It includes antibodies against 224 proteins related to cell signalling and reference proteins for standardisation in duplicate spots. Several variations of the protocol for different material sources are provided by the manufacturer, as no literature is available for an application to follicular fluids, and also because these fluids contain large amounts of albumin, the serum protocol was followed. As a consequence, the follicular fluids had to be stripped of the bulk protein present in these samples. Proteo-Prep® immunoaffinity columns for albumin and IgG depletion (Cat. No. PROTIA) were obtained from Sigma as recommended, and the provided instructions followed. Briefly, 100 *μ*L of FF containing 55.5 ± 7.9 mg/mL (mean ± SD) total protein (determined by Bradford assay) was loaded onto the column. In the collected effluents of 0.2 to 0.3 mL, the protein concentration was 2.60 ± 0.41 mg/mL. Fulfilling the requirements for running the arrays, the sample volumes were adjusted to 300 *μ*L containing 2.47 ± 0.36 mg/mL total protein (minimum requirement 1 mg/mL) and transferred to the Centre for Functional Genomics and Bio-Chips at the Institute of Biochemistry (Faculty of Medicine), University of Ljubljana, Slovenia, on dry ice. Cy3 and Cy5 dyes were purchased from LKB, Vienna, Austria. Each of the 20 matched-pair samples was labelled once with the Cy3 and once with the Cy5 dye. This reverse-dye approach is a convenient method of normalisation against label-specific interaction differences in array experiments. Each matched pair therefore underwent hybridisation once with the Cy3 label on the NC-IVF and the Cy5 on the cIVF sample and once the other way round, thus yielding a true, normalised duplicate. Labelling and hybridisation procedures were performed according to the manufacturer's protocol and materials (Sigma-Aldrich, CSAA1 Technical Bulletin [15]). The running of the experiment as well as the analysis of the results was performed on the machine at the same institute in Ljubljana using the software dedicated to this approach and also provided by the manufacturer (Sigma). The same method and analysis were applied, and the results described elsewhere in an analogous comparison between two patient groups [16]. The procedure included further normalisation algorithms; in our study, five of the 20 matched pairs were used to establish these algorithms and the results were proper calculated from the remaining 15 pairs. All signals pertaining to protein targets were between those internally provided on the arrays for positive and negative control. Housekeeping proteins included 90 spots (of which 45 proteins of the cytoskeleton), and the smoothing was applied over all spots with protein down weighting.

### 2.4. Specific Quantitation by ELISA

For the five out of 179 examined FF proteins for which an unadjusted *P* value of <0.05 was observed in both array duplicates for differential expression between NC-IVF and cIVF (three with NC>c and two with NC<c, see Results), an ELISA assay for their quantification was performed. We used commercial microplate ELISAs manufactured by Cloud-Clone Corporation, Wuhan, China, and available from Brunschwig Chemie, Basel, Switzerland, using antibodies different from those used in the antibody array. For these analyses and in contrast to the protein array experiment, the pairing was not necessary. For this reason, all follicular fluids with a sufficient available volume were included. These numbers varied between 35 and 37. The assay for human Cystatin A (Cat. No. SEA476Hu) had a functional sensitivity of 0.156 ng/mL, and all tested follicular fluids (*N* = 37, three had been used up in the array experiment) yielded a value above this figure. The assay for glutamic acid decarboxylase (GAD-2, Cat. No. SEB258Hu, corresponding to GAD65/67 on the array) came with a sensitivity of 0.1 ng/mL; one of the 37 unknown FF samples yielded a signal below this level. The assay for extracellular signal-regulated kinase-1 (ERK-1) (Cat. No. SEB357Hu) which belongs to the mitogen-activated protein (MAP) kinase family had a detection level of 0.118 ng/mL, but 18 FF samples yielded signal below this limit. The Caspase-3 assay (Cat. No. SEA626Hu) had a functional sensitivity of 0.114 ng/mL, but most FF samples (33 of 37) were found to contain lower amounts of this analyte. Similarly, the assay for the other MAP kinase ERK-2 (Cat. No. SEA930Hu) was not sensitive enough for the analysis of follicular fluids (34/37 below the detection limit of 2 pg/mL). The analysis of the results obtained by ELISA was therefore limited to the three markers Cystatin A, GAD-2, and ERK-1. Intra-assay coefficients of variance for all five assay kits were below 10%. All samples were assayed in one run/one plate according to the manufacturer's instructions. Nontreated FF sample aliquots (i.e., not albumin depleted as done prior to the array hybridisations) were diluted 1 : 3 in the provided diluent. A few samples required a repeat experiment for Cystatin A at a higher dilution (1 : 50). The results were analysed with Graphpad Prism® version 5.

### 2.5. Statistical Analysis

Statistical analysis was by nonparametric Wilcoxon signed rank test for paired samples. Correlation analysis was done using the paired Wilcoxon test. The association between detectable ERK-1 and the appearance of clusters in the MDS analysis was assessed by Fisher's exact test in a 2 × 2 contingency table.

## 3. Results

### 3.1. Patients

Mean patient age was 34.6 ± 3.1 (SD) years, and mean FF storage time was (at -80°C) 22.4 ± 14.9 months. Dependent of the selection process, these values did not statistically differ between the two groups, neither did the diameter of the follicle (the “leading follicle” in the stimulated cycle) at the end of the follicular or stimulation phase, which was 19.3 ± 2.4 mm over all 40 measurements (see Table 1). Serum estradiol levels, influenced by the administration of gonadotrophins, were 845 ± 256 pM in the NC-IVF and 8630 ± 4120 pM in the stimulated (cIVF) group (*P* < 0.0001).

### 3.2. CSAA1 Arrays

**Figure 1:**
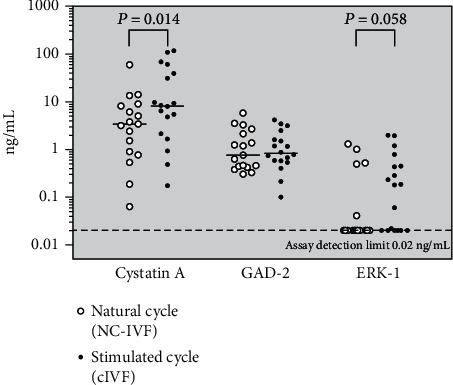
Follicular fluid levels of Cystatin A, GAD-2, and ERK-1 as determined by ELISA. Open circles, NC-IVF. Closed circles, cIVF (conventional stimulation with gonadotrophins). *P* values were obtained by the Wilcoxon signed rank test which takes the efficient pairing into account (the difference in the values between both cycles in the same patient, 15 sample pairs). The graph also includes the results from samples for which the pairing had not been possible due to an insufficient available volume. Please note the logarithmic scale.

**Figure 2:**
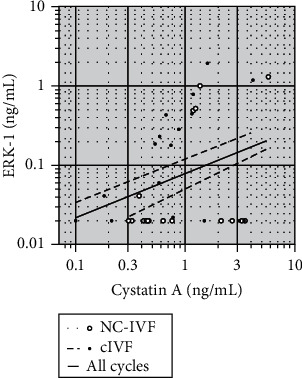
Correlation between the follicular fluid levels of MAP kinase ERK-1 and Cystatin A, as determined by ELISA, in all available samples. Open circles, NC-IVF. Closed circles, cIVF. Please note the double logarithmic scale. Regression lines are shown for the two stimulation protocols as well for the combined group. For *P* values, see text.

The analysis of the follicular fluid samples, performed over 15 matched NC-IVF/cIVF pairs after extensive internal normalisation using a large number of spotted reference, mostly cytoskeleton proteins, yielded a small number of differentially expressed markers amongst the remaining 179 “unknown” proteins in the array: applying a nonadjusted *P* value of 0.05, seven showed a higher and eight a lower expression level in the NC-IVF when compared to cIVF treatment cycle in one or both label combination replicates. These are presented in Table 2 as a function of increasing *P* values. The raw table contained another 482 lines (individual spots of proteins and all types of controls) with unadjusted *P* values >0.05 (not shown). The full array data table returned with all 179 “unknown” proteins (controls and structural proteins removed) and sorted by name is given in the Supplementary section (Table S1). After the adjustment of *P* values as a consequence of the multiple determinations, no significant differential expression could be observed for any of the markers available on the array. Table 2 also summarises the array results for the selected markers as a function of the unadjusted *P* value in either single or crossed-over labelling, including the direction of the differential expression (up or down in NC-IVF versus cIVF). Multidimensional scaling (MDS) analysis as returned by the software consistently yielded a distinct grouping of the samples into two clusters, irrespective of the number of proteins included over the array, ranging from the top 10 to the top 200. The distribution of the samples between the two clusters, however, was not associated with the IVF protocol used (natural or stimulated). An association between the distribution into these two clusters and the detection of MAP kinase ERK-1 above the 0.02 ng/mL detection limit (Figures 1 and 2) was nevertheless observed. Amongst the 16 samples located in cluster A, one had a detectable and 12 a nondetectable ERK-1 level (3 samples were not available for ERK-1 quantitation). Amongst the 13 samples in cluster B, eight had a detectable and three a nondetectable ERK-1 (2 samples not available). This association was statistically significant (Fisher's exact test, *P* = 0.002, quantity control samples excluded). As a consequence of the otherwise poor association between MDS clustering and the IVF stimulation protocol, this analysis was not continued.

### 3.3. Postarray Quantitation of Selected Marker Proteins by ELISA

A small number of markers, selected as a function of the returned array results but also of the availability of the corresponding immunoassays (specific antibodies), were quantified in the nontreated FF (the fraction obtained from the IVF laboratory after clarification by centrifugation but before protein depletion on the PROTIA columns). As stated in the Materials and Methods, the assays for Caspase-3 and MAP kinase ERK-2 did not present the required sensitivities. The results for the three other selected markers Cystatin A, GAD-2, and ERK-1 are shown in Figure 1. Cystatin A and MAP kinase ERK-1 levels were found to be significantly higher in the stimulated than in the natural cycles while glutamic acid decarboxylase 2 (GAD-2) did not differ in concentration between the two types of IVF stimulation and treatment (paired nonparametric sign test). Cystatin A and MAP kinase ERK-1 did not reveal a positive correlation between each other, with significance levels of *P* = 0.110 and *P* = 0.170 for the nonstimulated and stimulated cycles, respectively. However, when taken together, irrespective of the type of IVF protocol (*N* = 36), the obtained *P* value was 0.058, showing a trend towards a positive correlation between each other. These correlations are illustrated in a double logarithmic fashion in Figure 2.

## 4. Discussion

Our study revealed that the FF concentration of most analysed signalling proteins is not different between women undergoing gonadotropin stimulation and those in natural cycles. Only five proteins such as glutamic acid decarboxylase, Cystatin A, the MAP kinase ERK, Caspase-3, and diphosphorylated JNK yielded a significant difference. However, the antibody array findings did not correspond to the specific additional measurements performed by ELISA. Cystatin A was downregulated in the cIVF group, while in the ELISA, this protein showed higher FF levels in the cIVF than in the NC-IVF group. For GAD-2 (GAD65/67), no difference was observed in the ELISA analysis between the two stimulation groups. The same discrepancy was observed for ERK-1. Caspase-3 and ERK-2 could not be quantified by ELISA.

The reason for the conflicting results might be the use of confirming ELISA antibodies which were different from those supplied by the company which produced the CSAA1 protein array. This might be a weakness which was unexpected at the initiation of the project, but could also be interpreted as a strength of the study. As the differences were only observed in the CSAA1 protein array before *P* value adjustment and as the differences for the same markers in the ELISA were only marginal (though significant for two of them), the overall significance of the opposite findings can be questioned.

Interestingly, conflicting results were also found in two other antibody-based studies in which the FF cytokine concentrations were analysed. Baskind et al. [4] compared 40 FF and plasma cytokine concentrations in 10 women undergoing NC-IVF and cIVF treatments by fluid-phase multiplex immunoassays. Bersinger et al. [5] performed a similar study in 13 women with 13 mainly different cytokines by Luminex xMAP multiplexing technology. LIF was analysed in both studies. Whereas Baskind et al. [4] described significantly higher LIF concentrations in FF and plasma from NC-IVF compared to cIVF treatments, Bersinger et al. [5] did not find any differences. Conflicting results were also found for interleukin (IL) 6, IL8, IL10, and IL18, analysed by Baskind et al. [4] in plasma and by Bersinger et al. [5] in serum. Baskind et al. [4] did not find any differences whereas Bersinger et al. [5] found significantly higher concentrations in the serum of women undergoing cIVF and in comparison to NC-IVF.

The conflicting results of these and our own studies, all based on protein array technology, could be due to either high variability of specific FF proteins or constituents of FF which affect the accuracy of antibody-based assays.

Fifteen (four in duplicate and 11 in single determinations) FF proteins were found to be differentially expressed between NC-IVF and cIVF by nonadjusted *P* value; however, this precisely corresponds to the false-positive rate in multiple testing, and for this reason, the adjusted *P* value had to be applied. According to the adjusted *P* values, no protein tested on the Panorama array was differentially expressed between the two stimulation protocols.

Even though this seemed to be disappointing as such, it better corresponded to our ELISA results except for Cystatin A and ERK-1. ELISA being a well-controlled quantitative determination method for proteins, we would suggest to investigate these two markers in the follicular fluid of a larger number of samples. We had decided on the perfect match between the two stimulation groups by using one cycle each in the same patient, which strongly reduced the number of available samples (patients) for clinical reasons.

The absence of any significant differential expression by adjusted *P* value can also be the consequence of the presence of a large number or structural and intracellular proteins on the array. These could be derived from the separation of the cumulus cells in the mature follicle, which has been described [17, 18]. The selected (Panorama) array was developed for the analysis of cell signalling proteins, which we expected to be important players during the hormone-dependent maturation process in the follicle.

The results presented in Figure 1, however, indicate that differences may exist at this level between the two stimulation groups. Moreover, the two mentioned MDS clusters show an association to one or the other stimulation procedure, which is statistically significant (*P* = 0.002 by Fisher's exact test) but not exclusive, i.e., some samples were found in the “wrong” cluster. We were unable to find any clinical feature or irregularity in these particular samples which may explain a hypothetical association with the opposite group. Even with a precise definition of the IVF stimulation groups and subsequent attribution of a cycle into one of these, this illustrates that we are not dealing with a “black and white” situation and that patient-to-patient variations may play an even more important role than anticipated.

Our analysis of the ELISA results revealed a borderline correlation between the concentrations of Cystatin A and MAP kinase ERK-1, the two proteins which were found to show different FF concentrations in NC-IVF and cIVF.

Extracellular signal-regulated kinase-1 (ERK-1), which belongs to the mitogen-activated protein (MAP) kinase family, plays a role in signalling cascades and in transmitting extracellular signals to intracellular targets [19]. The activation of the ERK/MAPK signalling pathway promotes proliferation and has antiapoptotic effects. Activating mutations of this pathway are the most abundant oncogenic factors across all cancer types [20].

Cystatin A, also called acid cysteine proteinase inhibitor (ACPI), is strongly expressed in several different healthy tissues but is also expressed in malignant cells such as neoplastic cells of Hodgkin's disease [21]. Its physiological function is still unknown.

We found a borderline correlation of the concentration of ERK-1 and Cystatin A which might be due to the proliferation activity of growing follicles. However, this is pure speculative. Any specific functions of ERK-1 and Cystatin A in the physiology of the follicle cannot be derived from the literature yet.

## 5. Conclusion

Overall, our study suggests that concentrations of most FF signalling proteins are not different in follicles following natural or gonadotropin-stimulated cycles. Therefore, it can be assumed that differences in follicular physiology are apparently not substantially based on differences in the concentration of signalling proteins. However, this interpretation needs to be taken with caution as our study also confirmed that protein-based arrays of FF need to be interpreted with great care as they might not have the required sensitivity to pick up subtle differences in concentration.

## Figures and Tables

**Table 1:** Patient's characteristics.

	NC-IVF, *n* = 20	cIVF, *n* = 20
Age (years)	34.6 ± 3.3	34.5 ± 3.1
Follicular diameter (mm)	18.8 ± 2.5	19.8 ± 2.3

Cause of infertility		
Andrological, *n*	14	
Tubar, *n*	5	
Others, *n*	1	

**Table 2:** Raw data of the first 15 (nonstructural) protein markers as returned by the software and sorted by the unadjusted *P* value as analysed by the CSAA1 Panorama® array. The original raw table included contained another 499 lines (individual spots of proteins and all types of controls) with unadjusted *P* > 0.25 (not shown). The *P* values shown are means between two and four spots.

Rank by *P* (unadjusted)	Protein name	NC-IVF>cIVF		NC-IVF<cIVF		Unadjusted *P* value		Adjusted *P* value	
		Ratio	Log10	Ratio	Log10	Mean	SD	Mean	SD
1	Caspase-3—active			0.977	-0.0103	0.0148	0.0173	0.593	0.151
2	Glutamic acid decarboxylase (GAD65/67)	1.072	0.0301			0.0187	0.0011	0.620	0.002
3	Cystatin A	1.040	0.0172			0.0236	0.0080	0.659	0.058
4	MAP kinase (ERK-1+ERK-2)	1.055	0.0231			0.0321	0.0022	0.700	<0.001
5	JNK (activated)			0.980	-0.0086	0.0467	0.0175	0.751	0.071
6	NF-*κ*B			0.974	-0.0113	0.0515	0.0598	0.731	0.159
7	Nicastrin			0.0983	-0.0074	0.0571	0.0346	0.772	0.101
8	Chk2			0.0983	-0.0074	0.0571	0.0322	0.772	0.101
9	Cdh1	1.017	0.0075			0.0686	0.0747	0.743	0.177
10	Nerve growth factor receptor			0.977	-0.0099	0.0789	0.0565	0.812	0.080
11	FAK (phospho pS772)			0.984	-0.0072	0.0919	0.1055	0.759	0.199
12	Cdk-7/cak			0.986	-0.0061	0.0988	0.0801	0.818	0.089
13	Aop-1	1.023	0.0099			0.1415	0.1954	0.693	0.292
14	DAP kinase	1.015	0.0065			0.1870	0.2442	0.768	0.211
15	Cyclin A	1.032	0.0137			0.2308	0.1754	0.787	0.240

## Data Availability

Data are available from the authors on request.

## Supplementary Materials

Supplementary materials Table S1: the full array data table of the CSAA1 Panorama® array with all 179 proteins (controls and structural proteins removed) and sorted by name.
