# Supplementary material for: Gonadotropin Stimulation Has Only a Limited Effect on the Concentration of Follicular Fluid Signalling Proteins: An Antibody Array Analysis
Source: Int J Reprod Med. 2021 Jan 27;2021:2906164. doi: 10.1155/2021/2906164 (PMC7857919; doi:10.1155/2021/2906164)
Supplement: Supplementary materials — Table S1: the full array data table of the CSAA1 Panorama® array with all 179 proteins (controls and structural proteins removed) and sorted by name. [file 2906164.f1.pdf]

Block	Column	Row	ID	Name	log(FC*)	AveExpr	P Value	adj. P Value
19	1	4	S4813	a1 syntrophin	-0.1711	7.54	0.2159	0.970
11	1	2	H9161	Acetyl & phospho Histone H3(Ac-Lys9,pS10)	0.0680	7.72	0.8352	0.988
11	4	2	H9286	Acetyl-Histone H3 (Ac-Lys9)	0.0215	7.13	0.8421	0.981
21	2	1	A8967	Amyloid Precursor Protein (APP)	0.0183	7.69	0.6303	0.981
21	4	1	A8717	Amyloid Precursor Protein (APP) C-terminal	0.0042	7.73	0.7526	0.988
12	3	1	A7674	Aop-1	0.0314	6.56	0.1415	0.693
9	1	3	A5968	Ap-1/cjun	0.0312	7.39	0.2038	0.960
1	2	1	A7549	Apoptosis Inducing Factor (AIF)	0.0090	7.78	0.4228	0.972
24	4	3	A4721	ARNO (Cytohesin 2)	-0.0253	6.74	0.2400	0.970
1	3	1	A4471	ARTS	-0.0099	6.50	0.7126	0.981
9	4	3	A4086	ATF2	0.0146	7.34	0.1475	0.949
1	4	3	B0431	Bcl-10	0.0071	7.69	0.7494	0.981
1	2	2	B9304	Bcl-x	-0.0195	7.34	0.6036	0.977
1	3	2	B9429	Bcl-xL	0.0101	6.86	0.8021	0.988
20	2	2	N7155	b-NOS	-0.1755	7.87	0.3395	0.972
20	3	2	N2280	b-NOS	0.3834	8.37	0.8022	0.988
24	1	1	S1436	b-Synuclein (PNP-14)	0.0403	5.97	0.3685	0.971
5	4	1	A5844	c-Abl	0.0016	6.91	0.9341	0.988
12	3	3	C1956	Calcineurin	0.0189	6.70	0.5913	0.976
12	2	4	C0931	Calmodulin	0.0009	7.46	0.2745	0.970
13	1	1	C4731	Calnexin	0.0159	7.29	0.6464	0.981
13	3	1	C2687	Calponin	-0.0051	6.86	0.6378	0.982
13	2	2	C4606	Calreticulin	-0.0042	7.43	0.5043	0.965
25	1	2	C6974	CAM Kinase IIa	-0.0005	7.43	0.8592	0.981
25	3	2	C2851	CAM Kinase IV (Ay-18)	-0.0175	7.47	0.2471	0.961
3	4	2	C1229	Caspase 10	-0.0107	6.58	0.4812	0.977
3	2	2	C8351	Caspase 10	-0.0050	7.56	0.8603	0.981
3	2	3	C1354	Caspase 11	-0.0019	7.74	0.8428	0.982
3	3	3	C7611	Caspase 12	-0.0020	6.84	0.9090	0.988
1	1	3	C9598	Caspase 3	0.0022	7.84	0.6572	0.981
1	2	4	C8487	Caspase 3 - Active	-0.0358	7.65	0.0148	0.593
2	4	1	C3392	Caspase 4	-0.0677	5.95	0.2476	0.923
2	2	1	C4481	Caspase 4	0.0106	6.41	0.6394	0.983
2	1	2	C6979	Caspase 5	-0.0161	6.75	0.3085	0.971

2	4	2	C7599	Caspase 6	-0.0046	7.64	0.7546	0.988
2	2	3	C7724	Caspase 7	0.0078	7.82	0.7000	0.981
2	1	4	C3101	Caspase 8	-0.0183	7.43	0.1086	0.842
2	4	3	C4106	Caspase 8	0.0319	6.75	0.2667	0.970
3	4	1	C4356	Caspase 9	0.0007	6.67	0.4605	0.977
3	2	4	C0715	Cathepsin D	-0.0327	5.85	0.3830	0.965
5	1	2	C0349	Cdc25	0.0107	7.17	0.7409	0.981
5	3	2	C7104	Cdc27	-0.0032	6.63	0.8845	0.988
5	2	3	C0224	CDC6	0.0103	7.49	0.4141	0.976
29	1	1	C6613	Cdc7 Kinase	0.0387	6.71	0.3943	0.972
5	3	3	C7855	Cdh1	0.0222	6.74	0.0686	0.743
5	1	4	C8218	Cdk4	0.0034	7.40	0.8414	0.988
6	2	1	C8343	Cdk6	0.0080	7.02	0.8547	0.988
6	4	1	C7089	Cdk-7/cak	-0.0212	6.73	0.0988	0.818
6	1	2	C9358	Chk1	-0.0267	6.96	0.0921	0.875
6	4	2	C9233	Chk2	-0.0288	6.95	0.0571	0.772
6	1	3	C3956	c-myc	0.0239	7.83	0.4758	0.977
6	3	3	M5546	c-Myc	-0.0093	6.60	0.7759	0.981
21	2	2	C5922	CNPase	-0.0014	6.68	0.2410	0.961
21	4	2	C8736	Cofilin	-0.0098	7.95	0.5019	0.977
25	3	1	C0978	CRK-L	-0.0019	7.29	0.8990	0.988
9	1	4	C5112	CUG-BP1	0.0145	7.11	0.3551	0.976
7	1	1	C4210	Cyclin A	0.0415	6.81	0.2308	0.787
7	3	1	C4710	Cyclin A	-0.0149	6.30	0.2365	0.968
7	2	2	C8831	Cyclin B1	-0.0050	7.17	0.8916	0.988
7	4	2	C7464	Cyclin D1	-0.0090	6.38	0.7255	0.981
7	1	3	C7339	Cyclin D2	0.0121	7.25	0.4271	0.976
7	4	3	C7214	Cyclin D3	0.0006	7.03	0.5202	0.977
4	1	1	C3095	Cystatin A	0.0496	6.59	0.0236	0.659
4	1	2	D2178	DAP-Kinase	0.0178	6.72	0.1870	0.768
4	3	1	D7810	DAXX	0.0721	6.97	0.1578	0.949
21	1	3	D0180	DOPA Decarboxylase	0.0277	6.23	0.5981	0.977
21	3	3	D8043	Dystrophin	0.0081	6.85	0.5881	0.981
6	2	4	E9026	E2F1	0.0154	7.20	0.7780	0.981
25	1	3	E3138	EGF receptor	0.0211	6.46	0.2888	0.961

20	4	3	N3893	e-NOS	-0.0346	7.88	0.1946	0.960
20	2	3	N2643	e-NOS	-0.1091	6.86	0.4039	0.972
20	1	4	N9532	e-NOS	-0.0066	7.48	0.7073	0.981
25	1	4	E1523	ERK5 (Big MAPK-BMK1)	0.0004	7.72	0.8573	0.981
25	3	3	E0521	Estrogen Receptor (ER)	-0.0067	7.82	0.5462	0.981
26	1	1	F2918	FAK (focal adhesion kinase)	0.0105	7.63	0.5436	0.981
26	4	1	F9051	FAK Phospho (pS772)	-0.0252	6.55	0.0919	0.759
26	1	2	F9301	FAK Phospho (pS910)	-0.0196	6.53	0.3408	0.976
26	1	3	F7926	FAK phospho (pY397)	-0.0314	6.14	0.4203	0.977
26	4	2	F8926	FAK Phospho (pY577)	-0.0433	6.21	0.5463	0.977
4	2	3	G6916	GADD 153 (CHOP-10)	0.0233	7.86	0.1862	0.914
24	2	4	G6666	GAP1	-0.0230	6.74	0.1143	0.903
21	1	4	G9038	Glutamate receptor NMDAR 2a	0.0234	7.20	0.4149	0.976
22	1	1	G5163	Glutamic Acid Decarboxylase (GAD65/67)	0.0967	7.50	0.0187	0.620
22	4	1	G2781	Glutamine Syntethase	0.0006	7.21	0.9431	0.988
26	4	3	G2791	GRB-2	-0.0185	6.60	0.5960	0.977
25	1	1	G6541	GRP1(ARNO3 Cytohesin-3)	0.0301	6.70	0.5512	0.981
10	2	1	H7161	HAT1 (Histone acetyltransferase)	0.0188	7.08	0.6534	0.983
10	4	1	H3284	HDAC 1 (Histone Deacetylase 1)	-0.0139	7.87	0.2398	0.961
10	1	2	H3159	HDAC 2 (Histone Deacetylase 2)	-0.0077	7.62	0.5829	0.977
10	4	2	H9536	HDAC 4 (Histone Deacetylase 4)	0.0031	7.32	0.7799	0.981
10	1	4	R3777	hnRNP M3-M4	-0.0008	6.80	0.9514	0.994
12	1	2	H5147	HSP 70	-0.0241	6.19	0.5575	0.981
12	3	2	H1775	HSP 90	0.0215	6.27	0.2959	0.885
26	1	4	I0505	IkB a	-0.0047	7.13	0.5766	0.976
20	3	1	N9657	i-NOS	0.0227	6.66	0.2432	0.961
20	1	1	N7782	i-NOS	-0.0011	8.40	0.6931	0.981
27	2	2	J4500	JNK	-0.0774	6.81	0.6278	0.982
27	3	2	J4750	JNK activated (diphosporylated JNK)	-0.0313	6.80	0.0467	0.751
22	2	3	K3513	KIF3A	0.0068	6.93	0.5505	0.976
28	4	1	M7927	MAP Kinase (ERK-1)	-0.0064	7.60	0.7597	0.981
28	1	1	M5670	MAP Kinase (ERK1+ERK2)	0.0724	7.68	0.0321	0.700
28	1	2	M8159	MAP Kinase activated (diphosphorylated MAPK)	-0.0182	6.68	0.0879	0.875
28	3	2	M7802	MAP Kinase activated phosphothreonine	-0.0062	6.67	0.5632	0.981
28	1	3	M3682	MAP Kinase activated phosphotyrosine	0.0049	6.42	0.6062	0.977

28	1	4	M3550	MAP Kinase activated protein kinase-2 (MAKAPK2)	-0.0060	7.32	0.8072	0.988
31	2	2	M3787	MAP Kinase Phosphatase-1 (MKP-1)	-0.0198	7.81	0.2225	0.960
28	3	3	M3807	MAPK non phosphorylated ERK	0.0533	6.31	0.1021	0.914
27	2	4	M8434	Mcl-1	-0.0070	7.64	0.7720	0.981
7	2	4	M7815	MDM2	-0.0138	6.89	0.6859	0.981
27	1	1	N2661	NAK	-0.0272	6.75	0.4194	0.976
12	1	3	N2786	Nedd 8	-0.0131	7.01	0.4141	0.972
22	4	2	N5408	Nerve Growth Factor Receptor	-0.0313	6.02	0.0789	0.812
22	1	2	N3908	Nerve Growth Factor Receptor (NGFR p75)	-0.0212	7.43	0.5292	0.981
23	4	2	N0142	Neurofilament 200	-0.0241	6.75	0.3989	0.977
23	2	2	N4142	Neurofilament 200	-0.0255	7.85	0.4586	0.976
27	4	1	N8523	NF-kB	-0.0391	6.63	0.0515	0.731
22	4	3	N1660	Nicastrin	-0.0266	7.45	0.0571	0.772
11	4	3	N9527	NTF2	-0.0251	6.68	0.1697	0.960
24	3	2	P1870	p120CTN	-0.0153	7.69	0.5609	0.981
8	1	1	P2610	p14 arf	0.0063	6.51	0.5661	0.977
8	3	1	P0968	p16INK4a/CDKN2	-0.0016	6.64	0.3190	0.961
8	1	2	P4354	p19INK4d	0.0310	6.62	0.2556	0.971
8	4	2	P1484	p21Waf-1	0.0030	7.49	0.7961	0.981
8	1	3	C3085	p34cdc2	0.0035	6.91	0.6129	0.977
8	4	3	P9489	p35	-0.0101	7.41	0.8287	0.981
27	2	3	M0800	p38 MAPK	-0.0071	7.51	0.5242	0.977
27	3	3	M8177	p38 MAPK activated (diphosphorylated p38)	-0.0147	6.71	0.3767	0.965
8	1	4	P5813	p53	-0.0164	7.59	0.1119	0.949
9	2	1	P2735	p57kip2	0.0195	6.81	0.3719	0.972
9	4	1	P3737	p63	-0.0084	7.41	0.5142	0.976
4	3	3	P5367	PAR4 (Prostate Apoptosis Response 4)	-0.0138	7.89	0.8208	0.988
10	4	3	P7493	PCAF	0.0461	7.12	0.2356	0.970
4	1	4	P1495	Phosphatidylserine Receptor (PSR)	-0.0070	6.86	0.9245	0.988
4	3	2	D4941	Phospho-DAPK (pS308)	0.0114	7.24	0.6196	0.977
11	1	1	H6409	Phospho-Histone H3 (pS10) Clone:H3-P	-0.0053	6.53	0.6458	0.977
11	3	1	H9908	Phospho-Histone H3 (pS28)	0.0036	7.16	0.7122	0.981
29	2	2	P5242	Phospholipase A2 group V	0.1301	8.36	0.6588	0.981
29	4	2	P8104	Phospholipase C g1	0.3593	7.31	0.0863	0.875
29	4	1	P3237	Phospho-PAK (pS212)	0.0063	6.71	0.7151	0.981

30	2	2	P4112	Phospho-PKB (pS473)	-0.0087	6.98	0.4184	0.977
30	3	2	P3862	Phospho-PKB (pT308)	-0.0099	6.95	0.6517	0.981
32	4	1	P7114	Phospho-Pyk2 (pY579)	0.0105	6.49	0.7373	0.981
32	2	2	P6989	Phospho-Pyk2 (pY579/580)	-0.0221	6.13	0.5278	0.977
32	3	2	P6739	Phospho-Pyk2 (pY580)	0.0108	6.35	0.6329	0.981
32	1	3	P6864	Phospho-Pyk2 (pY881)	0.0029	6.34	0.9179	0.988
32	2	4	R1151	Phospho-Raf (pS621)	0.0072	6.59	0.4813	0.976
9	2	2	R6878	Phospho-Retinoblastoma (pS795)	-0.0065	6.75	0.4382	0.977
29	2	3	P3430	Phosphoserine	0.1346	7.69	0.1486	0.949
24	1	2	T6819	Phospho-Ta (pS199/202)	-0.0128	6.15	0.7591	0.981
29	1	4	P3555	Phosphothreonine	0.0368	7.84	0.6023	0.981
29	3	3	P1869	Phosphotyrosine	0.0591	6.94	0.1925	0.960
30	2	1	P2482	PKB /AKT	0.0021	6.15	0.7798	0.981
30	3	1	P1601	PKB/AKT	-0.0101	7.36	0.5113	0.977
30	2	3	P4334	PKC a	0.0080	6.99	0.7615	0.981
30	3	3	P3203	PKC b	-0.0086	6.61	0.9024	0.981
31	1	1	P8083	PKC g	0.0086	6.91	0.7367	0.981
30	2	4	P3328	PKC g	-0.0001	6.46	0.8563	0.981
31	3	1	P3987	PKD	-0.0021	7.37	0.8118	0.988
3	1	1	C7849	Procaspase 8	0.0036	7.19	0.8348	0.988
31	3	2	P7979	Protein phosphatase 1a	-0.0140	7.16	0.0976	0.869
31	2	3	P7482	PTEN	-0.0153	7.52	0.1608	0.910
31	4	3	P3487	PTEN	-0.0127	6.33	0.5254	0.977
32	2	1	P3902	Pyk2	0.0355	7.33	0.4299	0.965
32	4	3	R5773	RAF1	-0.0064	7.20	0.6472	0.977
11	2	3	R4777	RAN	-0.0150	7.09	0.6574	0.981
23	4	1	S2407	S-100	-0.0580	7.24	0.6972	0.981
23	1	1	S2532	S-100 b	-0.0137	6.03	0.7115	0.981
31	2	4	S5188	SGK (serum and glucorticoid inducible kinase)	-0.0062	7.46	0.4105	0.977
5	2	1	S0941	SMAC/DIABLO	0.0375	7.58	0.2153	0.915
9	3	2	S3934	SMAD4	0.0584	7.61	0.3334	0.972
22	2	4	S9684	SNAP-25	-0.0043	7.63	0.6013	0.977
10	2	3	S8316	SUV39H1 Histone Methyl Transferase	0.0228	6.65	0.4108	0.972
23	1	3	S2177	Synaptotagmin	-0.0180	7.41	0.1579	0.949
23	4	3	S0664	Syntaxin	-0.0535	6.52	0.3754	0.972

23	2	4	S3062	Synuclein a	-0.0193	7.25	0.1274	0.789
11	2	4	T8573	Topoisomerase-1	-0.0130	6.88	0.7576	0.988
12	1	1	T1948	Trf-1	0.0243	6.71	0.5951	0.981
24	2	3	T0678	Tryptophane Hydroxylase	0.0033	6.20	0.7922	0.981
24	4	1	T2928	Tyrosine Hydroxylase	-0.0057	6.80	0.8667	0.988

*** Fold Change**
